# KLF4 Alleviates Hypertrophic Scar Fibrosis by Directly Activating BMP4 Transcription

**DOI:** 10.7150/ijbs.71167

**Published:** 2022-05-09

**Authors:** Jing Wang, Ming Zhao, Hongyun Zhang, Fangfang Yang, Liang Luo, Kuo Shen, Xujie Wang, Yan Li, Jian Zhang, Jinxin Zhang, Kejia Wang, Jin Li, Yunwei Wang, Yunshu Yang, Dahai Hu

**Affiliations:** 1 Department of Burns and Cutaneous Surgery, Xijing Hospital, Fourth Military Medical University, 127 Changle West Road, Xi'an, Shaanxi 710032, China;; 2 State Key Laboratory of Military Stomatology, National Clinical Research Centre for Oral Diseases, Shaanxi International Joint Research Centre for Oral Diseases, Department of Oral Anatomy and Physiology and TMD, School of Stomatology, Fourth Military Medical University, 127 Changle West Road, Xi'an, Shaanxi 710032, China; 3 State Key Laboratory of Cancer Biology and National Clinical Research Centre for Digestive Diseases, Xijing Hospital of Digestive Diseases, Fourth Military Medical University, 127 Changle West Road, Xi'an, Shaanxi, 710032, China; 4 Department of Emergency, Xijing Hospital, Air Force Medical University, 127 Changle West Road, Xi'an, Shaanxi 710032, China

**Keywords:** Krüppel-like factor 4, Hypertrophic scar, Transcriptional activation, BMP4

## Abstract

**Background:** Hypertrophic scars (HS) often occur after burns, surgery and extensive trauma. Krüppel-like factor 4 (KLF4) is a member of the Krüppel-like factor family, a group of conserved zinc finger transcription factors that regulate diverse cellular processes. KLF4 can participate in the regulation of fibrotic diseases in many organs, such as the lung, liver, and heart. However, the antifibrotic effect of KLF4 in skin HS remains elusive.

**Result:** This study observed the inhibition of KLF4 on fibrosis *in vivo* and *in vitro*. Our results revealed that KLF4 expression was decreased in HS tissue and fibroblasts. The results of KLF4 transfection confirmed its ability to alleviate the transdifferentiation of fibroblasts into myofibroblasts both *in vitro* and *in vivo*, thereby inhibiting the development of fibrosis. In addition, ChIP assays showed that BMP4 was the target gene of KLF4 for inhibiting skin fibrosis.

**Conclusions:** Collectively, this evidence indicates that KLF4 is associated with BMP4 and could play an important regulatory role in HS formation by downregulating myofibroblast transdifferentiation. Our study provides a new target for the prevention and treatment of hypertrophic scars.

## Introduction

Hypertrophic scar (HS) is one of the most common sequelae of patients after burns or trauma, and it develops in up to 70% of patients with burns 1. HS is a kind of skin lesion caused by excessive hyperplasia repair of new connective tissue after damage to the dermis or deep tissue, and it is often accompanied by symptoms that include pain, itching and even loss of function. HS can lead to serious physical and mental effects on patients and cause physical and psychosocial disorders, which reduce the patients' quality of life and hinder their reintegration into society. Approaches to minimizing scar formation, including optimizing the healing potential of wounds, have been widely applied. However, an ideal treatment that leads to complete scar prevention has not been possible. Hence, the molecular mechanisms underlying scar formation and its prevention must be further investigated.

As a serious fibrotic skin disease, HS is caused by abnormal wound repair processes, in which fibroblasts play a crucial role. In the process of wound healing, fibroblasts are responsible for wound contraction as well as collagen synthesis and secretion. In response to wound stimulation, fibroblasts express α-SMA and transdifferentiate into myofibroblasts. Transformation from fibroblasts to myofibroblasts increases the synthesis and secretion of collagen I and collagen III, which are major extracellular matrix components in human skin and regulate cell migration, proliferation and specific gene expression. However, this trauma-initiated response is also the pivotal process in HS formation 2 since activated myofibroblasts can lead to disturbances in collagen metabolism. Therefore, blocking the activation of myofibroblasts is an effective means of preventing HS 3.

Krüppel-like factor 4 (KLF4) is a member of the Krüppel-like factor family, a group of conserved zinc finger TFs that regulate diverse cellular processes, such as cell growth, proliferation and differentiation 4. Interestingly, KLF4 has also been reported to be involved in the process of fibrosis, and it has been reported to suppress myofibroblast activation and inhibit fibrosis in chronic kidney disease 5. In the renal tubules of diabetic animals, KLF4 expression is significantly reduced while TGF-β expression is increased. KLF4 can regulate renal fibrosis by inhibiting the TGF-β-induced release of proinflammatory cytokines 6. Similarly, KLF4 could exert protective effects against lung fibrosis 7 and corneal fibrosis 8 by suppressing TGF-β-induced fibroblast-to-myofibroblasts. Meanwhile, the levels of α-SMA, TGF-β and Col1 are significantly increased in hepatic stellate cells (HSCs) after silencing KLF4 9. However, the effect of KLF4 on the inhibition of fibrosis in skin has never been reported.

In this study, we revealed that the expression of KLF4 in HS-derived fibroblasts (HSFs) was lower than that in normal skin-derived fibroblasts (NSFs). When the expression of KLF4 was upregulated in HSFs, fibrosis-related molecules, including Col1, Col3, and α-SMA, were downregulated. Subsequently, a bleomycin-induced fibrosis mouse model was introduced, and it revealed that KLF4 significantly reduced the extent of fibrosis. Furthermore, RNA-seq showed that the expression of BMP4 was proportionally related to KLF4 levels, which was corroborated by ChIP-PCR and sequencing assays showing that KLF4 targeted BMP4. When the expression of BMP4 was knocked down via siRNA, the regulatory effect of KLF4 on fibrosis-related molecules was abolished. Consistently, when the expression of BMP4 was upregulated by BMP4Δ, the regulatory effect of KLF4 was restored. This evidence indicates that KLF4 could play an important regulatory role in HS formation by downregulating myofibroblast transdifferentiation.

## Results

### Expression of KLF4 was downregulated in HSFs

**Figure 1:**
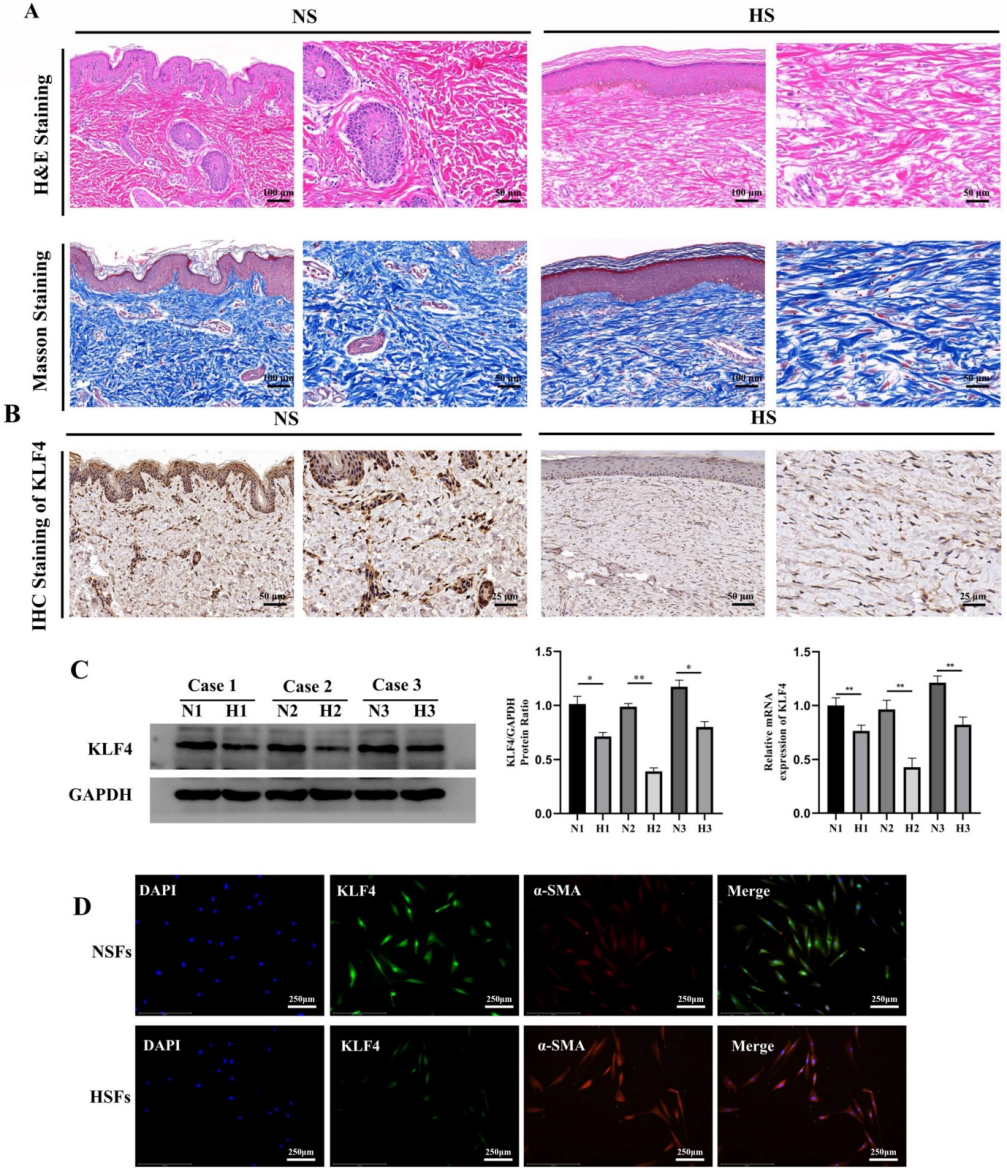
** KLF4 was dramatically downregulated in HS.** (A) H&E and Masson staining of NS and HS originating from patients with HS. (B) IHC staining for KLF4 in NS and HS tissues from patients with HS. (C) Western blot and PCR for KLF4 in three paired NS and HS tissues. (D) Immunofluorescence double staining for KLF4 and α-SMA in NSFs and HSFs. *P < 0.05 and **P < 0.01 compared with the NS tissues.

We collected HS skin tissues and normal full-thickness skin tissues from three patients. H&E and Masson trichrome staining showed that the dermal thickness in HS was thicker than that in normal skin. In addition, HS tissue displayed excessive, tense and disorderly arranged collagen fibres while normal skin tissue displayed a thinner and ordered collagen arrangement (Figure 1A). Subsequent IHC staining showed that KLF4, which was significantly downregulated in HS tissues compared with NS tissues, was mainly expressed in the nucleus of fibroblast cells in the dermal layer, with occasional expression in the cytoplasm (Figure 1B). To further determine the expression of KLF4 in fibroblasts derived from NS and HS tissues, mRNA and protein samples were extracted from HSFs and NSFs for qRT-PCR and western blot assays (Figure 1C). The results showed that KLF4 was decreased at both the mRNA and protein levels in HSFs compared with NSFs (Figure 1C). Moreover, immunofluorescence double staining performed on both HSFs and NSFs indicated that KLF4 was remarkably reduced in myofibroblasts with significant α-SMA expression. In contrast, obviously high levels of KLF4 protein were detected in α-SMA-negative NSFs, especially in the nucleus (Figure 1D). Collectively, these results demonstrated that the expression of KLF4 was downregulated in both HS tissues and HSFs, thus indicating that KLF4 might be involved in the transdifferentiation of fibroblasts in HS.

### KLF4 dampened fibrosis-related molecules in HSFs

**Figure 2:**
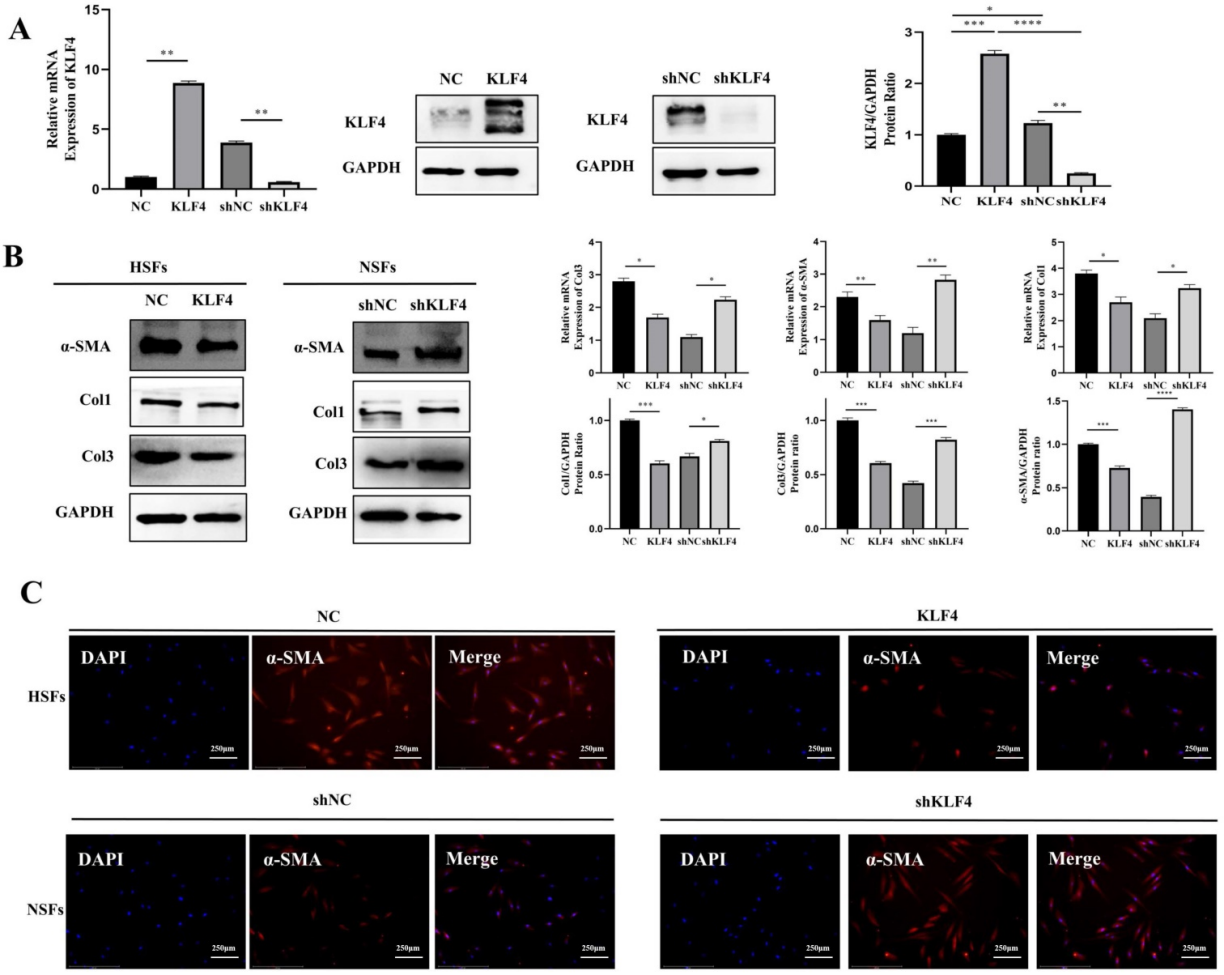
** KLF4 significantly dampened fibrosis-related molecules in HS fibroblasts.** HSFs were infected with KLF4-overexpressing lentivirus or negative control vector, and NSFs were infected with the shKLF4 or control shRNA lentiviral vector. (A) KLF4 mRNA and protein levels were evaluated after infection in the indicated cells. (B) Fibrosis-related molecules, including α-SMA, Col1 and Col3, were analysed by qRT-PCR and western blot in the indicated cells. (C) Immunofluorescence staining for α-SMA in the indicated cells. *P < 0.05, **P < 0.01, ***P < 0.001 and ****P < 0.0001 compared with the negative control.

To verify the regulatory effect of KLF4 on fibrosis, we constructed a KLF4 overexpression vector and shRNA to exogenously alter the expression of KLF4 in HSFs and NSFs. Specifically, HSFs were infected with the KLF4-expressing (KLF4 group) or empty control vector (NC group), and KLF4 in NSFs was knocked down with the shKLF4 (shKLF4 group) or control shRNA lentiviral vector (shNC group). Based on qRT-PCR and western blotting, KLF4 expression was significantly enhanced in HSFs and inhibited in NSFs (Figure 2A). The detection of fibrosis-related molecules, including Col1, Col3 and α-SMA, showed that ectopic KLF4 overexpression resulted in conspicuously reduced expression of fibrosis-related proteins (Figure 2B). In contrast, NSFs with silenced KLF4 exhibited significantly increased expression of Col1, Col3 and α-SMA at both the mRNA and protein levels (Figure 2B). Meanwhile, immunofluorescence staining of α-SMA showed that KLF4 overexpression in HSFs robustly downregulated α-SMA while KLF4 knockdown significantly upregulated α-SMA (Figure 2C). Collectively, these results indicated that KLF4 can reduce the expression of fibrosis-related proteins.

### KLF4 inhibited the proliferation of HSFs

**Figure 3:**
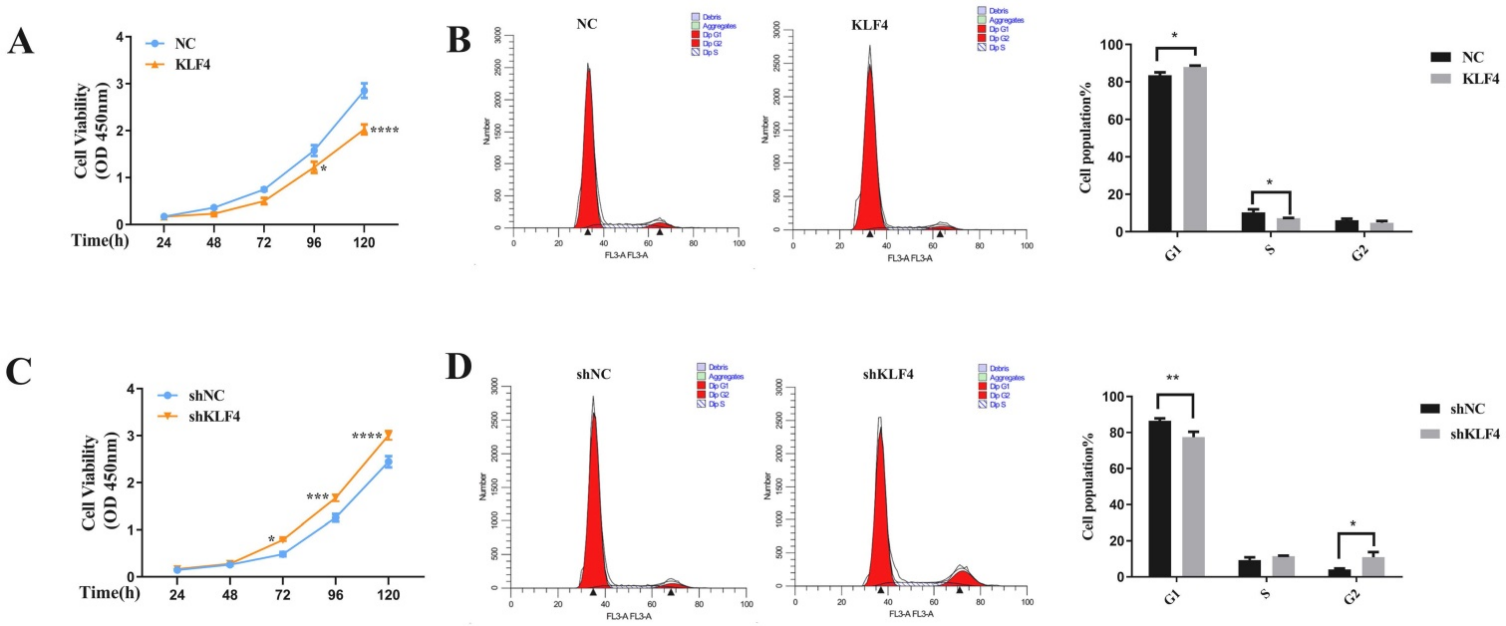
** KLF4 significantly inhibited the proliferation of HS fibroblasts via cell cycle arrest.** (A & C) CCK-8 assays were used to determine cell viability in the indicated HSFs infected with KLF4 or NC lentiviral vector and NSFs infected with shKLF4 or control shRNA lentiviral vector. (B & D) Cell cycle distributions were detected by flow cytometry. *P < 0.05, **P < 0.01 and ***P < 0.001 compared with the negative control.

Next, our CCK-8 assays showed that KLF4 upregulation inhibited HSFs proliferation while KLF4 downregulation significantly promoted NSFs proliferation (Figure 3A & 3C). To further illustrate the growth inhibitory effect of KLF4, the cell cycle was measured by flow cytometry, and the results showed that KLF4 upregulation increased the G1 phase ratio in HSFs (Figure 3B) while KLF4 downregulation decreased the G1 phase ratio in NSFs (Figure 3D). In summary, KLF4 greatly inhibited the proliferation of HSFs by inducing G1 phase arrest.

### KLF4 alleviated hypertrophic scar *in vivo*

**Figure 4:**
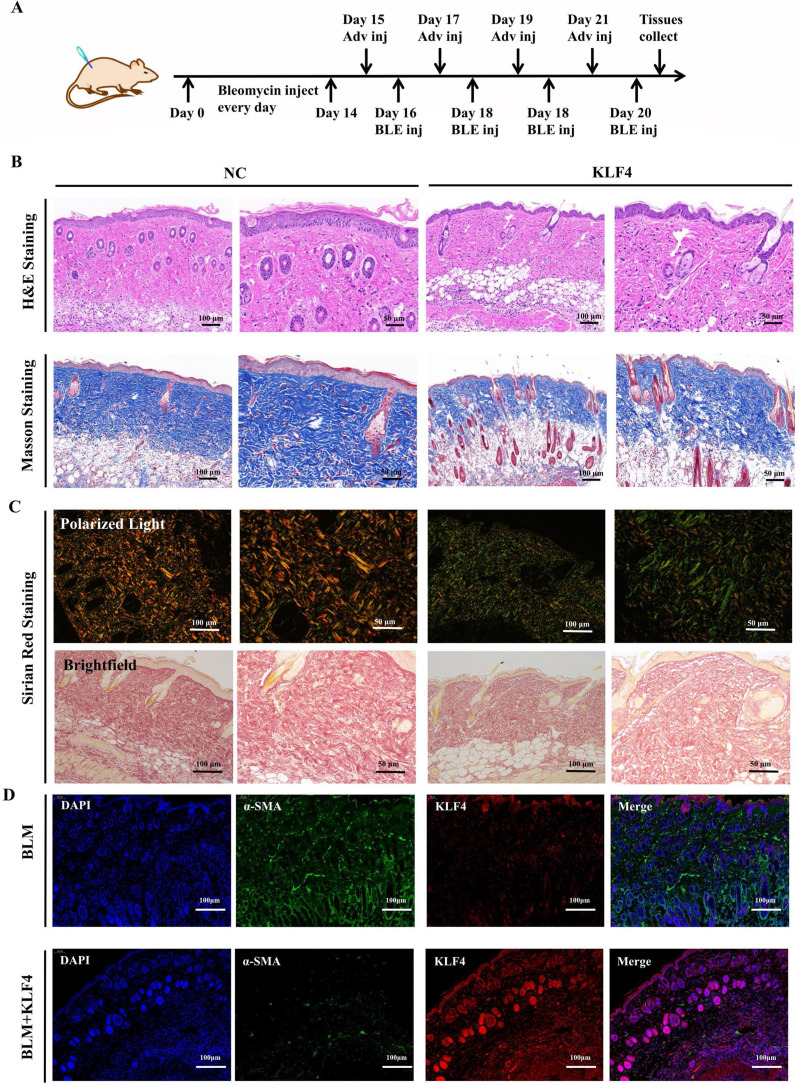
** KLF4 dramatically alleviated hypertrophic scar *in vivo.*** (A) Bleomycin was persistently injected into BALB/c mice for 2 weeks, and then KLF4/NC adenovirus was injected into the region of bleomycin-induced dermal fibrosis in mice. (B) H&E and Masson staining were performed with HS tissues from the KLF4-overexpressing and NC groups. (C) Sirius Red staining was performed with HS tissues from the KLF4-overexpressing and NC groups. (D) Immunofluorescence double staining for KLF4 and α-SMA in HS skin tissues. (E) Immunofluorescence staining for Ki67 in HS skin tissue.

To verify the antifibrotic effect of KLF4 *in vivo*, a fibrosis model was constructed via persistent bleomycin injection into the dermis of BALB/c mice. After dermal scarring was successfully established, KLF4/NC adenovirus was injected into the region of bleomycin-induced dermal fibrosis in mice. At 21 days after modelling, dermal tissues were collected for subsequent testing (Figure 1A). H&E and Masson's trichrome staining showed that KLF4 overexpression in bleomycin-induced fibrotic skin resulted in a thinner epidermis and dermis, less collagen deposition and a more ordered arrangement of collagen structures (Figure 4B). Sirius Red staining and polarized light microscopy were conducted to observe the distribution of Col1 and Col3. The results revealed that the ratio of Col1 (red or yellow) was lower than that of Col3 (blue) in the KLF4-overexpressing fibrosis model while the ratio of Col1 was higher than that of Col3 and distributed more densely in the control group (Figure 4C). Moreover, immunofluorescence double staining of HS skin tissues for KLF4 and α-SMA showed that the KLF4 intervention directly targeted α-SMA-positive fibroblasts, thus demonstrating that KLF4 could effectively reduce the numbers of myofibroblasts of HS (Figure 4D). Additionally, immunofluorescence staining for Ki67 revealed that KLF4 inhibited the proliferation of HSFs *in vivo*. Thus, our results demonstrated that KLF4 could improve the collagen structure arrangement and proportion and decrease α-SMA-positive myofibroblasts in a bleomycin-induced fibrosis mouse model.

### BMP4 is a key downstream target of KLF4

**Figure 5:**
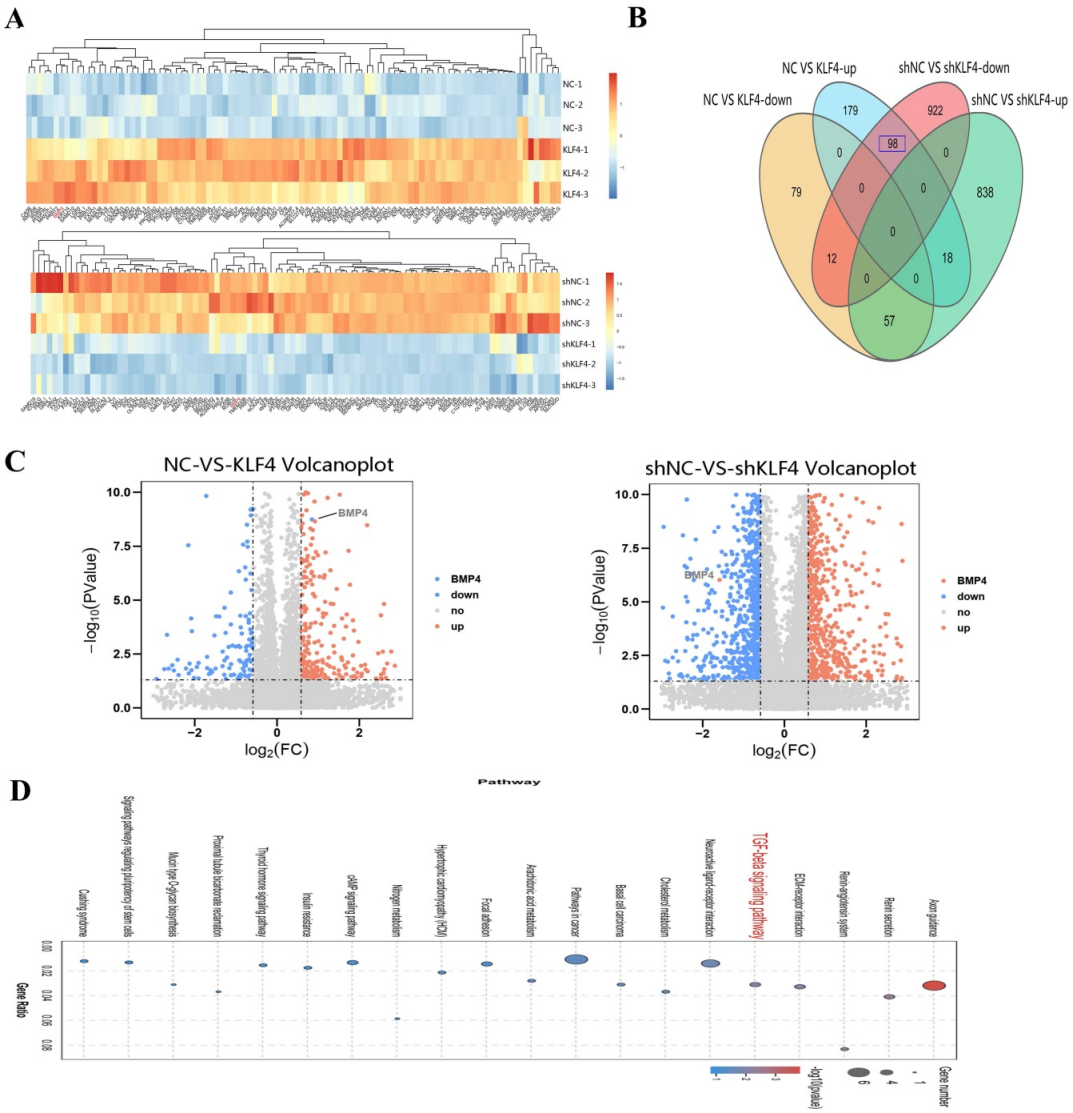
** BMP4 was selected as a key downstream target of KLF4 via RNA sequencing.** (A) Heatmap for the visualization of a clustering analysis of differentially expressed genes (DEGs). (B) Venn diagram of the overlap number of DEGs between comparisons. (C) DEGs, differentially expressed genes. (C) Volcano plots showing the upregulated (red) and downregulated (blue) DEGs for each comparison. BMP4 was clearly marked. (D) KEGG pathway network analysis of differentially expressed genes.

To further reveal the role of KLF4 in HS, RNA sequencing was performed to search for the downstream genes directly regulated by KLF4. Transcriptional expression differences between KLF4 and NC in HSFs and between shNC and shKLF4 in NSFs are shown in Figure 5A. Compared with HSFs infected with the NC empty lentivirus, as many as 443 genes were differentially expressed in HSFs infected with the KLF4-overexpressing lentivirus, among which 255 genes were upregulated and 148 were downregulated (*|FC|* ≥ 1.5, *P* < 0.05). Moreover, 1945 genes were differentially expressed between the shNC and shKLF4 groups of NSFs, among which 913 genes were upregulated and 1032 were downregulated (*|FC|* ≥ 1.5, *P* < 0.05). A conjoint analysis of the two groups indicated that as many as 98 genes showed the same expression trend in both the NC vs. KLF4 group and shNC vs. shKLF4 group (Figure 5B). Considering that KLF4 functions greatly in regulating multiple cellular processes as an imperative transcription factor, we analysed the possible downstream targets of KLF4 via further bioinformatics analyses. KEGG pathway association analysis indicated that several fibrosis-related pathways, including ECM-receptor interaction and TGF-β signalling pathways, were significantly upregulated in the selected gene set. Interestingly, the expression of BMP4, a key molecule in the TGF-β signalling pathway, was proportionally correlated with the expression of KLF4 (Supplementary Figure 1). As shown in the volcano plot, the expression of BMP4 increased significantly in the NC vs. KLF4 group but decreased significantly in the shNC vs. shKLF4 group (Figure 5C). Consequently, transcriptome sequencing and further bioinformatics analysis indicated that BMP4 might be a promising target of KLF4 to regulate several fibrosis-related signalling pathways.

### KLF4 directly activated BMP4 transcription

**Figure 6:**
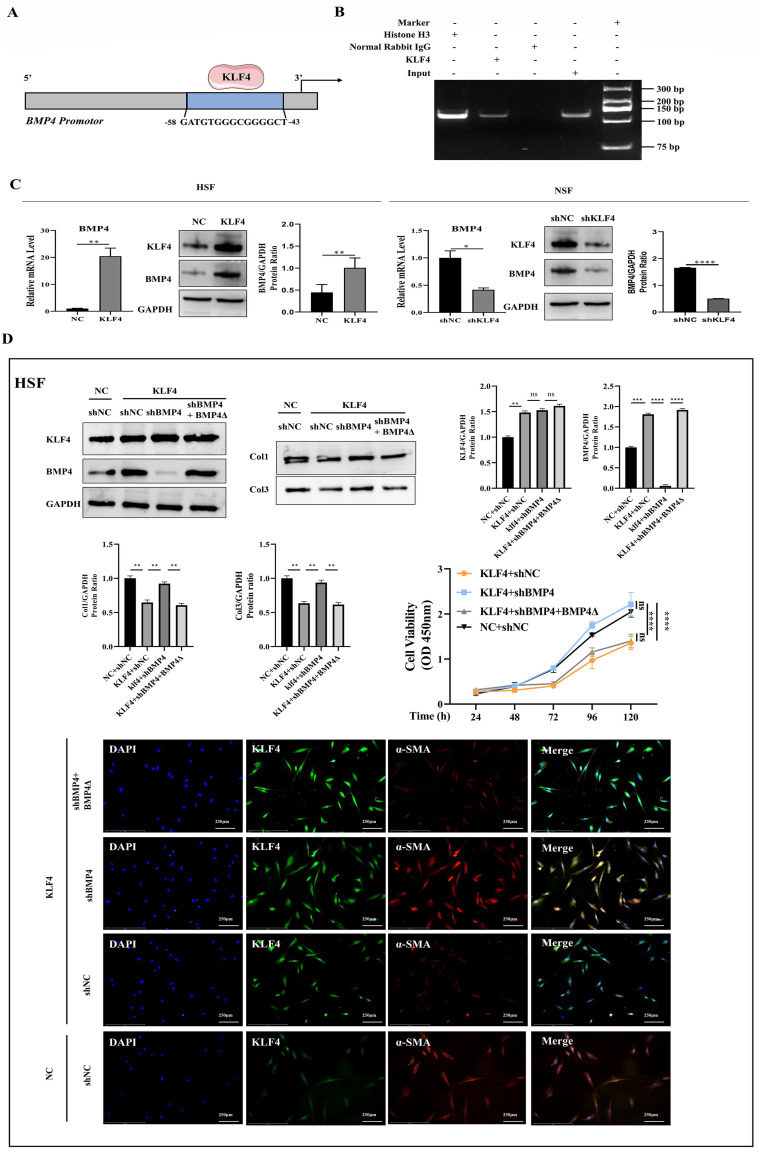
** KLF4 positively regulated BMP4 transcription.** (A) Schematic diagram of the BMP4 promotor. The blue region is the putative KLF4-binding site. (B) Amplification of the BMP4 promoter sequence from chromatin immunoprecipitation (ChIP) DNA was performed. Histone H3, IgG and input served as the positive, negative and internal controls, respectively. (C) qRT-PCR and western blot for BMP4 in HSFs infected with KLF4 lentiviral vector or NC vector and NSFs infected with shNC or shKLF4 lentivirus. (D) Downregulated BMP4 in HSFs inhibited proliferation and fibrosis-related molecule expression induced by KLF4 overexpression. A shBMP4-resistant reconstitution, BMP4Δ, was used to restore BMP4 expression, which reversed the fibrosis of HSFs. *P < 0.05, **P < 0.01, ***P < 0.001 and **** *P* < 0.0001 compared with the negative control.

To forecast the KLF4-binding region on the *BMP4* promotor, TRANSFAC (http://bioinfo.life.hust.edu.cn/AnimalTFDB/#!/tfbs_predict) was used as the data for the bioinformatics algorithm. The bioinformatics analysis revealed that the promotor region of the *BMP4* gene (-58 to -43) contained a KLF4-binding sequence (5'-GATGTGGGCGGGGCT-3') (Figure 6A). To confirm this hypothesis, ChIP-PCR was performed, and the results verified that KLF4 bound to the *BMP4* promotor (Figure 6B). The upregulation of KLF4 in HSFs resulted in the increased expression of BMP4, while the downregulation of KLF4 in NSFs by the shKLF4 lentivirus decreased the expression of BMP4 (Figure 6C). Furthermore, BMP4 downregulation in HSFs rescued the inhibited cell proliferation and expression of fibrosis-associated proteins induced by KLF4 overexpression. A mutant shBMP4-resistant lentiviral vector was then used to restore BMP4 expression, which resuppressed the proliferative and profibrotic ability of HSFs (Figure 6D). These findings suggest that BMP4 could serve as a key target gene in HS proliferation and fibrosis induced by KLF4 overexpression.

## Discussion

The most prominent feature of hypertrophic scars (HS) is the excessive accumulation of extracellular matrix (ECM) during tissue repair, which is due to the excessive proliferation and secretion of fibroblasts 1. Although fibroblasts are quiescent in normal skin tissue, they can be activated by trauma or inflammation and polarized to myofibroblasts secreting collagen and fibronectin 10. It is widely accepted that polarization of fibroblasts to myofibroblasts is the key process in fibrosis, and it involves several signalling pathways and key molecules; moreover, α-smooth muscle actin (α-SMA) is a marker of myofibroblast polarization, and it is associated with a higher capacity for ECM synthesis and scar contracture 11, 12. An increasing number of signalling pathways are reported to be involved in fibrosis, including TGF-β/SMAD, Wnt/β-catenin and YAP/TAZ. However, the mechanism of HS is still unclear, thus rendering existing treatment far from satisfactory 13. Hence, further elucidation of the molecular mechanisms underlying scar formation and prevention is paramount.

KLF4 is an important component of the KLF transcription factor family, which regulates various biological processes, such as cell proliferation, differentiation and self-renewal 14, 15. An increasing number of studies have reported that KLF4 can regulate fibrosis in various organs by inhibiting the transdifferentiation of fibroblasts into myofibroblasts 16. KLF4 is reported to regulate the TGF-β pathway in fibroblasts and inhibit the proinflammatory differentiation of macrophages by regulating TNF-α. Although several studies have indicated that activation of YAP by KLF4 can promote chronic renal function decline and the development of interstitial fibrosis 17, the role of KLF4 in skin fibrosis has rarely been reported. Consequently, the relationship between KLF4 and HS calls for further investigation. In this study, the comparison between HS and normal skin revealed that the expression of KLF4 in HS and HSFs was lower than that in NS and NSFs. Reports have indicated that the overexpression of KLF4 in several cell lines, including a human colon cancer cell line, induces cell cycle arrest by blocking G1/S progression 18. We revealed that highly expressed KLF4 inhibited the proliferation of HSFs by inducing G1 phase arrest. Overexpression of KLF4 in HSFs alleviated the expression of fibrosis-related molecules, including Col1, Col3 and α-SMA, while inhibition of KLF4 in NSFs boosted the expression of fibrosis-related molecules.

In our *in vivo* experiments, we discovered that KLF4 upregulation effectively alleviated the extent of dermal fibrosis (particularly collagen deposition and dermal thickness) in an animal model of bleomycin-induced skin fibrosis. This evidence strongly confirmed the antifibrotic effect of KLF4 *in vivo*. However, due to phylogenetic differences between different species, considerable debate remains about the appropriate animal model for fibrosis 19. Compared with humans, mouse skin has several distinct characteristics, including thick hair coverage and rapid growth rate, no dermal papilla or sweat glands, and no hypertrophic scars during the healing process 20. Moreover, while human hypertrophic scars can be transplanted into immunodeficient mice, the process of human hypertrophic scar formation, which is perceived as the final stage of scar formation, cannot be simulated due to the absence of immune function in nude mice 21, 22. Although the characteristics of hypertrophic scars in Duroc pigs are similar to those in humans, this large animal model has a number of shortcomings, such as high cost, strict feeding conditions and difficult handling 23. Many studies have shown that bleomycin can directly activate a series of proinflammatory and profibrotic factors that play an important role in the scarring process in humans, such as TGF-β, PDGF, and IL-1β 24-26. Bleomycin-induced skin fibrosis in mice has pathological features similar to human hypertrophic scars, including inflammatory cell infiltration, skin thickening by collagen deposition, disorganized collagen, and absence of skin appendages 27. However, *in vivo* models of bleomycin-induced hypertrophic scarring are also problematic. First, the role of restoring epidermal integrity in inhibiting hypertrophic scar formation could not be replicated, which can alleviate skin fibrosis after burn injury 28. In addition, the cause of hypertrophic scarring is mainly excessive fibroblast proliferation and deposition of extracellular matrix caused by chronic inflammation; however, bleomycin does not induce chronic inflammation.

As a versatile transcription factor, KLF4 is involved in regulating numerous target genes. To clarify the regulatory effect of KLF4 on skin fibrosis, we detected downstream genes regulated by KLF4 via transcriptome sequencing for KLF4- and NC-overexpressing HSFs as well as shNC- and shKLF4-infected NSFs. Further bioinformatics analysis revealed that fibrosis-related pathways, such as TGF-β and ECM-receptor interactions, were regulated by KLF4. Interestingly, BMP, a group of signalling molecules that belong to the TGF-β superfamily, was regulated by KLF4. KLF4 regulates various biological processes by targeting the promoters and enhancer regions of certain genes and recruiting active histone marks to these sites. A bioinformatics algorithm predicted that BMP4 could be a theoretical target of KLF4, and ChIP-PCR revealed that KLF4 could directly bind to the promoter of BMP4 and activate the transcription of BMP4. BMPs regulate multiple organogenetic processes, embryonic development patterns, and adipose and energy metabolism 29, 30. BMP4 can modulate TGF-β signalling pathways via two specific BMP receptors on target cells 31, 32. The coordinated activation of members of the BMP/TGF-β signalling molecule family, which recruits osteogenic and chondrogenic progenitor cells, promotes the development of calcific cardiovascular disease 33. BMP4 significantly increases collagen production in cardiac fibroblasts and induces the differentiation of fibroblasts into myofibroblasts in cardiac fibrosis. Another study indicated that BMP4 can block TGF-β2-stimulated ECM production in trabecular meshwork (TM) cells, while in primary open-angle glaucoma (POAG), gremlin may antagonize the natural role of BMP4 in TM cells 34. Several studies have indicated the potential of BMP4 to induce adipogenic transformation of scar fibroblasts. Plikus MV et al. 35 reported that BMP4 facilitated the reprogramming of myofibroblasts to adipocytes. Overexpression of the BMP4 antagonist Noggin or abolishment of the BMP4 receptor in myofibroblasts prevented adipocyte formation. Another study found that myofibroblast reprogramming was triggered by BMP4 and that activation of PPARγ signalling initiated tissue remodelling. Recombinant human BMP4 was added to TGF-β-treated myofibroblasts and led to distinct α-SMA downregulation. BMP4 promoted SMAD1/5/7 phosphorylation, thereby inducing PPARγ activation. The expression of the MAPKs ERK and p38 was inhibited by PPARγ, and the profibrotic effect of TGF-β was antagonized in myofibroblasts^36^. In this study, by inhibiting and restoring BMP4 expression in HSFs overexpressing KLF4, we revealed that the inhibitory effect of KLF4 on fibrosis was eliminated when the expression of BMP4 was silenced. BMP4 is the downstream target of KLF4, and the regulatory effect of KLF4 on fibrosis depends on the expression of BMP4.

## Conclusion

**Figure 7:**
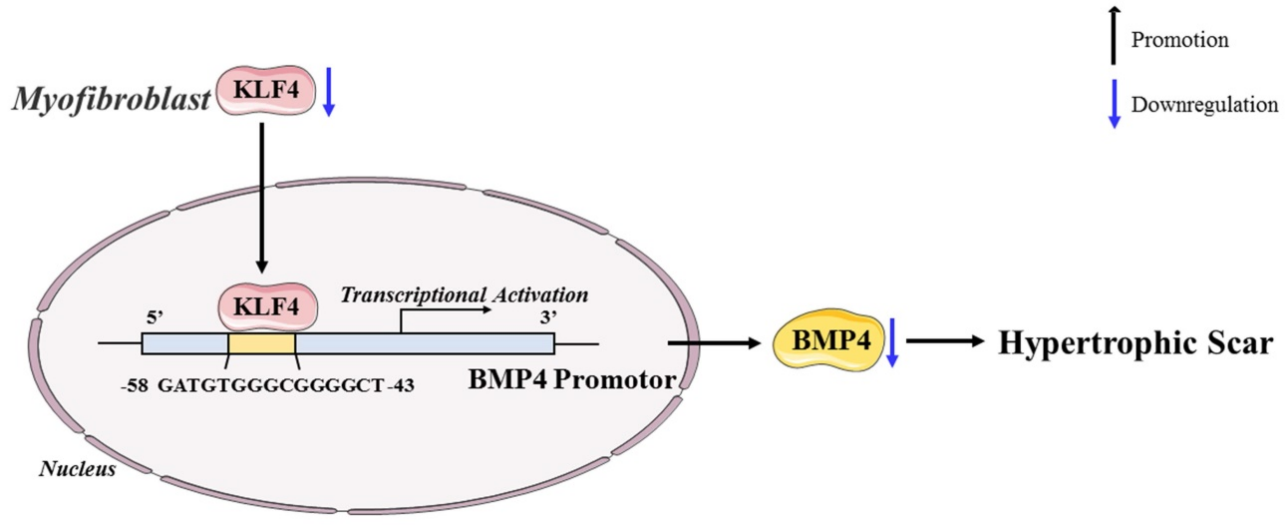
Schematic diagram of the proposed mechanism.

In conclusion, our study revealed the antifibrotic role of KLF4 in HS and the mechanisms by which KLF4 inhibits HS fibrosis by directly activating BMP4 transcription (Figure 7). The findings inspire a novel therapeutic idea and elucidate the particular mechanism underlying the clinical treatment of HS. Accordingly, we anticipate that KLF4 might become a rational indicator of dermal fibroblast transdifferentiation and a promising target for HS gene therapy.

## Methods and Materials

### Patients and ethics statement

Three pairs of normal skin (NS) and hypertrophic scars (HS) tissues were collected from patients. Before surgery, the patients were notified of the purpose and procedures of the study and agreed to participate in this study. Written informed consent was obtained from all patients or their legal guardians, and this study was approved by the Ethics Committee of Xijing Hospital, Fourth Military Medical University.

### Isolation and culture of HSFs and NSFs

The dermal portions of hypertrophic scar tissues and normal skin tissues were minced and cultured in collagenase type Ⅰ (0.1 mg/ml) at 37 °C for 3 hours to isolate fibroblasts. HS-derived fibroblasts (HSFs) and NS-derived fibroblasts (NSFs) were incubated with DMEM with low glucose (31600034, Gibco) supplemented with 10% FBS (35-081-CV, Corning), 100 U/ml penicillin, and 100 ug/ml streptomycin. Third- to fifth-passage HSFs and NSFs were used for the subsequent experiments.

### Real-time quantitative polymerase chain reaction (qRT-PCR)

Total RNA was extracted using TRIzol reagent (9108, Takara) and quantified to confirm the concentration. Then, cDNA was generated using a Prime Script^TM^ RT reagent kit (RR037A, Takara). qPCR was conducted using Ultra SYBR Mixture (CW0957H, CWBIO) and specific primers on a Bio-Rad IQ5 Real-Time System (Bio-Rad). A 10 μl volume was subjected to the PCR regimen: 95 °C for 10 min, 40 cycles of denaturation at 95 °C for 15 s, and 60 °C for 1 min. The expression levels of target genes were normalized against GAPDH. Forward 5′ -GCACCGTCAAGCTGAGAAC-3′ and reverse 5′-TGGTGAAGACGCCAGTGGA-3′ for human GAPDH, forward 5′-CTCAGGGTGTCAAGGGTGAAAGTG-3′ and reverse 5′-TGTACCAGCCAGACCAGGAAGAC-3′ for human Col1, forward 5′-CTCAGGGTGTCAAGGGTGAAAGTG-3′ and reverse 5′-GCCAGCTGCACATCAAGGAC-3′ for human Col3, forward 5′-TCGTGCTGGACTCTGGAGATGG-3′ and reverse 5′-CCACGCTCAGTCAGGATCTTCATG-3′ for human α-SMA, forward 5′-ACTGGGACGGCTGTGGATGG-3′ and reverse 5′-CTTCATGTGTAAGGCGAGGTGGTC-3′ for human KLF4, forward 5'-CGTAGCCCTAAGCATCACTCACA-3'; reverse 5'-GCGCCGGCAGTTCTTATTCT-3 for human BMP4.

### Western blot analysis

Cell pellets were lysed with RIPA buffer (AR0102, BOSTER) with protease inhibitor added on ice. The protein concentration was determined according to the instructions of the Pierce™ BCA Protein Assay Kit (23227, Thermo). Equal amounts of protein were loaded onto 10% SDS-PAGE gels, separated by electrophoresis and transferred onto PVDF membranes (IPVH00010, Millipore). After blocking with 5% skim milk in TBST for 1.5 hours at room temperature, the membranes were incubated with rabbit anti-human Col1 (ab138492, Abcam), rabbit anti-human Col3 (ab184993, Abcam), rabbit anti-human KLF4 (ab214666, Abcam), rabbit anti-human α-SMA (ab7871, Abcam), rabbit anti-human BMP4 (ab124715, Abcam), and rabbit anti-human GAPDH (60004-1-lg, Proteintech) antibodies overnight at 4 °C. The next day, the membranes were incubated with IgG-HRP secondary antibody (A0208, Beyotime) for 1 h at 37 °C. Traces of immunoreactivity on the membrane were detected by ECL reagent (WBULS0100, Millipore) on a Fluor Chem FC system (Alpha Innotech). Bands were quantitated by ImageJ software.

### Flow cytometry analysis

The cell cycle was measured by flow cytometry. Fibroblasts were harvested, and precooled 75% ethanol was added and cryopreserved at -20 °C for 2 h. Afterwards, ethanol was discarded, and the cells were washed with PBS at room temperature, resuspended in propidium iodide (PI) DNA staining buffer and incubated for 15 min at room temperature in the dark. The cell cycle in each phase was checked on a flow cytometer (FACSAria, BD Biosciences).

### Cell Counting Kit-8 (CCK-8) assay

CCK-8 (CK04, Dojindo Laboratories) assays were performed according to the manufacturer's protocol. Treated cells were inoculated into a 96-well plate at a density of 3,000 cells/well with three replicate sets and then incubated at 37 °C for 24 h. The next day, 10 μl CCK-8 solution and 90 μl of complete medium were added to each well. After incubation at 37 °C for 1 h, the absorbance was measured at 450 nm with a microplate reader to detect the number of viable cells.

### Animal model

A bleomycin-induced dermal fibrosis model was established with 6-week-old male BALB/c mice. The BALB/c mice were purchased from the Laboratory Animal Centre of the Fourth Military Medical University (Xi'an, China) and fed normal food and water on a 12-hour light/dark cycle. First, mice received daily dorsal subcutaneous injection with bleomycin (100 μg/100 μl, dissolved in PBS) (n = 12). Two weeks later, bleomycin was injected once every two days (n = 12). On the other days, KLF4-overexpressing adenovirus (constructed by HanBio) was injected subcutaneously daily (n = 6). As a control, the other six mice were injected with the same amount of empty control vector (n = 6). After one week, the mice were sacrificed and skin tissues were obtained for subsequent experiments.

### Histopathology, picrosirius red and Masson trichrome staining

The tissues were fixed with paraformaldehyde, dehydrated with graded ethanol, and embedded in paraffin. The samples were cut into 5 μm thick slices, which were used for Masson, haematoxylin and eosin (H&E) and picrosirius red staining. Briefly, specimens from three animals were deparaffinized in 100% xylene, washed twice in 100% ethanol and PBS, and then immersed in picrosirius red (Sirius Red 0.1% in picric acid) at room temperature for 1 h. After washing with PBS, the sections were dehydrated immediately, cleared in xylene and mounted. Collagen fibres were detected by light and polarized light microscopy. Under polarized light microscopy, Col1 fibres were stained red while Col3 fibres appeared green.

### Immunofluorescence staining

Cells grown on coverslips were fixed with 4% paraformaldehyde for 20 min. After fixation, Triton X-100 (93443, Sigma Aldrich) was used to permeabilize the membrane for 15 min and then blocked with 1% BSA for 30 min at room temperature to inhibit nonspecific binding of IgG. In terms of double-labelling immunofluorescence staining for KLF4 and α-SMA, the slips were incubated in primary antibodies against rabbit anti-human KLF4 and antibodies against mouse anti-human α-SMA overnight at 4 °C. The next day, the antibody was discarded, and the slips were then incubated with DyLight 488-conjugated goat anti-rabbit IgG (A23220, Abbkine) and IFKine red-conjugated donkey anti-mouse IgG (A24411, Abbkine) antibodies for 1 h at room temperature in the dark. For single-label immunofluorescence to detect α-SMA, the primary antibody was mouse anti-human α-SMA and the secondary antibody was IFKine red-conjugated donkey anti-mouse IgG. Nuclei were stained with DAPI Fluoromount-G (Southern Biotech, Birmingham, AL, USA; Catalogue No. 0100-20). Images were taken with an FSX100 microscope (Olympus).

### Establishment of stable KLF4 overexpression and KLF4 knockdown cell lines

The KLF4-overexpressing and shRNA lentiviruses were constructed by Shanghai GeneChem Co., Ltd. HSFs and NSFs were plated onto six-well plates until reaching 60-70% confluence and then infected with KLF4-overexpressing or KLF4 shRNA lentivirus separately at a multiplicity of infection (MOI) of 100 for 12 h according to the manual.

### Transcriptome sequencing and analysis

RNA extraction, library construction and sequencing. Total RNA was extracted using TRIzol reagent (9108, Takara) following the manufacturer's protocol. RNA quality was evaluated on an Agilent 2100 Bioanalyzer (Agilent Technologies) and checked by RNase-free agarose gel electrophoresis. After total RNA extraction, eukaryotic mRNA was enriched by Oligo (dT) beads. Then, the enriched mRNA was fragmented into short fragments using fragmentation buffer and reverse transcribed into cDNA with random primers. Second-strand cDNA was synthesized by DNA polymerase I, RNase H, dNTPs and buffer. Then, the cDNA fragments were purified with a QiaQuick PCR extraction kit (Qiagen), end repaired, poly (A) added, and ligated to Illumina sequencing adapters. The ligation products were size selected by agarose gel electrophoresis, PCR amplified, and sequenced using Illumina Novaseq6000 by Gene Denovo Biotechnology Co. (Guangzhou, China).

### Chromatin immunoprecipitation (ChIP)

ChIP was monitored using a SimpleChIP® Enzymatic Chromatin IP Kit (9003, Cell Signaling Technology) according to the manufacturer's protocol. Cells were cross-linked in 1% formaldehyde at 37 °C for 10 min and washed with PBS 3 times. Nuclear proteins were extracted in 1 M dithiothreitol, and DNA was digested into fragments of 150-900 bp by micrococcal nuclease and subsequent sonication. The nuclear extracts were then centrifuged at 9,400×g, and the supernatants containing cross-linked chromatin were collected. The recovered supernatants were incubated with KLF4 antibodies (11880-1-AP, Proteintech) at 4 °C overnight and then with ChIP-Grade Protein G Magnetic Beads (9006, Cell Sirabbit IgG (2729, Cell Signaling Technology) were used as the positive control and negative control, respectively. After the chromatin preparations were eluted and decrosslinked with 5 M NaCl and 5 mg/ml Proteinase K at 65 °C for 2 h, the DNA was purified and used for PCR with the BMP4 ChIP forward primer TCGGGCGCTGCCTGG and reverse primergnaling Technology) for 2 h. Histone H3 (D2B12) XP® rabbit mAb (4620, Cell Signaling Technology) and normal CTCCTTCCCTCCTCCCTCG according to the binding sequence TCGGGCGCTGCCTGGGCTTCCGGGACCCGGGCCTGCTAGGCGAGGTCGGGCGGCTGGAGGGGAGGATGTGGGCGGGGCTCCCATCCCCAGAAAGGGAGGCGAGCGAGGGAGGAGGGAAGGAG.

### Antagonism and rescue-of-function experiment of BMP4

An antagonism and rescue-of-function experiment was designed using shBMP4 to knock down BMP4 in KLF4-overexpressing HSFs, and a shBMP4-resistant reconstitution, BMP4Δ, to restore BMP4 expression. To prevent shBMP4 from counteracting the restoration of BMP4 expression, a synonymous mutation technique was employed to silence the shBMP4-targeting sequence “CCCTGGTCAATTCTGTCAATT”, which is located in the CDS of the BMP4 gene (transcript: NM_001347912.1). The mutated sequence is “CCTTGGAGCATCCTGAGCATC”.

### Statistical analysis

All data were analysed using GraphPad Prism 8.0. Every experiment was repeated at least three times, and the data are presented as the mean ± standard error of the mean. Statistical analyses were performed by Student′s t tests and two-way ANOVA. *P* < 0.05 was considered statistically significant.

## Supplementary Material

Supplementary figure.

## Abbreviations

HS: hypertrophic scars
NS: normal skin
KLF4: krüppel-like factor 4
HSCs: hepatic stellate cells
HSFs: hypertrophic scars derived fibroblasts
NSFs: normal skin derived fibroblasts
CCK-8: cell counting kit-8
H&E: haematoxylin and eosin
Col1: collagen I
Col3: Collagen III
ChIP: chromatin immunoprecipitation
DEGs: differentially expressed genes
