# Supplementary material for: KLF4 Alleviates Hypertrophic Scar Fibrosis by Directly Activating BMP4 Transcription
Source: Int J Biol Sci. 2022 May 9;18(8):3324–36. doi: 10.7150/ijbs.71167 (PMC9134901; doi:10.7150/ijbs.71167)
Supplement: Supplementary file 1 — Supplementary figure. [file ijbsv18p3324s1.pdf]

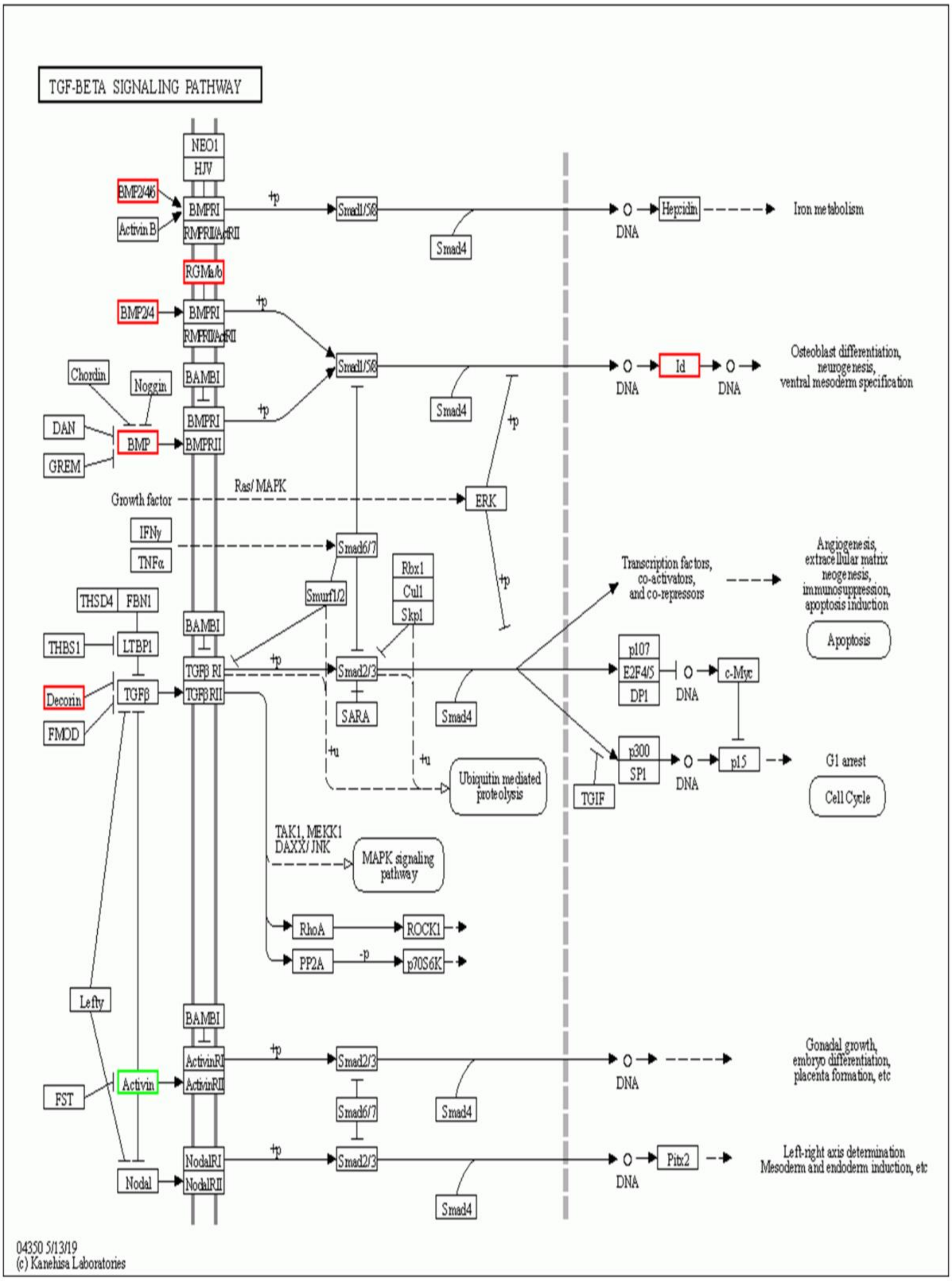

Supplementary Fig KEGG enrichment analysis of TGF-β signaling pathway-related molecular labeling maps
